# Social Distancing, Safe Spaces and the Demand for Quarantine

**DOI:** 10.1007/s12115-020-00500-8

**Published:** 2020-07-19

**Authors:** Frank Furedi

**Affiliations:** The University of Kent, Canterbury, CT2 7NY UK

**Keywords:** Social distance, Safe space, Personal boundary, Fear

## Abstract

Social distance has been a topic of interest in sociology for more than a century before the outbreak of the COVID-19 pandemic. Whereas in the past it referred to the distance between groups in more recent times it signifies the space between individuals. The aspiration for safe space and personal boundaries in recent years indicated that social distancing has acquired an increasingly individuated and privatised form. This article suggests that the demand for safe space can be interpreted as a demand for a quarantine from psychic threats. This pre-existing demand for a quarantine from criticism and pressure has seamlessly meshed with the imperative of social distancing in the COVID-19 era.

As a sociologist I was astonished to discover that so many people have become comfortable with their life in lockdown. When in the course of a zoom conference in May 2020, I tell a colleague in New York that I am going stir crazy and want my life back, she admonishes me for thinking irresponsible thoughts. Another attempts to reassure me that there is much that I can do “to make life more comfortable” during the lock down.

One major survey published on the UK in May 2020 indicated that 49% of the respondents agreed with the statement that “there are some aspects of the “lockdown” measures that I’ve enjoyed.”^1^ Surveys also suggest that millions of people are worried that the lockdown is being eased too rapidly.^2^ Numerous commentators claim that social distancing is here to stay. These responses to a public health crisis did not emerge out of nowhere. They have a prehistory and what this essay will attempt to show is that what the COVID-19 pandemic has done is to amplify and reinforce a pre-existing cultural orientation towards the spatial dimension of safety.

## Social Distance

It is useful to recall that the term social distance has its origins in the field of sociology. The first significant sociological contribution to distancing is to be found in Georg Simmel’s seminal essay, “The Stranger” in 1908. Simmel’s essay stimulated the development of the concept of social distance in the field of American urban sociology in the 1920s. Emory Bogardus, founder the Department of Sociology, University of South Carolina developed the social distancing scale, which was mainly used to measure inter-racial relations and different forms of “prejudice”. At this point in time social distance was primarily used to explore the physical and psychical distance between groups.

From the standpoint of today, arguably the most interesting contribution to understanding the cultural dynamics surrounding social distancing is to be found in the work of Karl Mannheim, one of the founders of the sociology of knowledge. Mannheim was interested in understanding the application of the concept of social distance to cultural life. At this point in time social distance did not refer to the physical distance between individuals but.to the social and cultural distance between groups in society. He pointed out, that throughout history, groups have sought to maintain a distance between themselves and other groups by establishing boundaries between themselves and others. Those others were sometimes deemed to be polluting as in the case of the caste system of India or simply as a threat to the safety to the people living behind the walled cities of feudal Europe.

Mannheim was probably the first sociologist to explore the relationship between social distance and what today is characterised as “safe space”. Writing in the 1930s and preoccupied by the threat of totalitarian movements, Mannheim referred to safe space in his discussion of social distance, which he claimed could signify both “an external or spatial distance” or an “internal or mental distance”. Mannheim believed that the impulse towards distancing was bound up with the need to regulate and control anxiety. He suggested that distancing is “one of the behaviour patterns which is essential to the persistence and continuity of an authoritarian civilisation”. Democracy on the other hand, he argued “diminishes distances”.^3^ It was in the context of the fears that emerged in the inter-war era that Mannheim located the aspiration for a safe space. He observed that “The evolution of mental distancing from spatial distance can be clearly demonstrated in the case of fear. If I keep a safe space between myself and the stranger who is stronger than me, then, this spatial distance between us there is contained in the mental distance of fear”.^4^ Mannheim perceived that the desire to keep a safe space between oneself and the stranger was closely related to anxiety and fear. But a fear of what? His proposition that the maintenance of a safe space was intimately connected to the fear of social degradation suggests that what was also at stake are anxieties about harms to identity.

Mannheim’s association of social distancing with the fears thrown up in an authoritarian setting can be understood as a statement about the prevalence of trust within a society. It is likely that today, the anxieties bred by mistrust have influenced attitudes towards social distancing and have also created a demand for safe spaces. It is also the case that some of the authoritarian impulses that Mannheim associated with the quest for safe space in the 1930s continue to influence the demand for it in in the contemporary era.

Since Mannheim’s exploration of social distancing, the metaphor of a safe space has turned into a cultural ideal that enjoys widespread institutional support. It is a metaphor that is widely adopted in the sphere of socialisation, childrearing, nurseries, schools and of course in higher education. Yet, despite its extensive usage the term itself is rarely explored or defined. What appears to be important about the metaphor of a safe space is that it speaks to a general aspiration for what the sociologist, Anthony Giddens calls ontological security- the sense of order and continuity – in the face of uncertainty.^5^

## Safe Space as an Inverted Quarantine

Inverted Quarantine is a concept developed by the sociologist Andrew Szasz in his study, *Shopping Our Way to Safety: How We Changed from Protecting the Environment to Protecting Ourselves.*^6^ Unlike a traditional quarantine, which seeks to isolate a disease to keep it spreading to the public, an inverted quarantine represents the opposite impulse of people isolating themselves from the harms that they perceive as threatening them. Inverted quarantine constitutes a response to the fear that the human condition is inherently unsafe. Arguably the most striking example of a demand for an inverted quarantine is the idealisation of a safe space and its proliferation. Whereas a gated community is designed to keep out undesirable outsiders, the purpose of a safe space is to protect its inhabitants from unwelcome criticism and thoughts.

It was in 2015 that media attention directed at the demand for safe spaces led to the widespread recognition of this practice. However, the demand for this form of inverted quarantine emerged a long time before campaigns for safe spaces exploded in Anglo-American universities. It was the concern with psychic survival that surfaced in the late 1970s that provided the initial impetus for the emergence of attitudes that eventually led to the formulation of the demand for safe space. This early relationship between psychic survival and safe space was captured in the title of the psychiatrist, Anthony Fry’s book, *Safe Space: How to Survive in a Threatening World*. For Fry, a safe space was necessitated by the perilous world that exists outside the self. He wrote that “as I looked carefully at this rather threatening world, it seemed that safe space for many of us was becoming increasingly hard to find and that for a whole variety of reasons, material, social and personal conditions were becoming ever more unsuitable for human beings”^7^ His ideal was what he described as the “protected spaces of childhood” and in many respects his metaphor of safe space captures the security of the child still in the womb.^8^

The concept of safe space highlights the most distinct feature of the way that contemporary culture conceptualises safety. The designation of certain spaces as safe implicitly suggests that what lies outside of it is likely to be unsafe. The absence of safety is the premise for the argument for safe spaces. During the lockdown that followed the outbreak of the pandemic perceptions about the absence of safety were heightened and spaces that could be considered safe contracted. This point was stressed by Jonathan Mayer, professor emeritus of geography and epidemiology at the University of Washington, who stated that as danger, real or perceived, creeps closer, the notion of safe space narrows, then narrows more “until it’s really anything outside the home”.^9^ From this standpoint everything outside our home is unsafe and a quarantine is a guarantor of a safe space and staying indoors offers security from inherently dangerous world.

But from what types of threats do safe spaces provide protection? Its idealisation on campuses and elsewhere did not emerge in response to a public health emergency like coronavirus. Typically, in the pre- COVID-19 era the demand for safe space was justified on the grounds that, since people are vulnerable and emotionally fragile, they need to be quarantined from the toxic effects of criticism and judgment. From this perspective the issue of personal safety is enmeshed with the provision of protection from words and ideas that are likely to further damage an individual’s identity.

The concerns voiced by safe space advocates was not so much directed at protecting public health but at threats directed at their psyche and identity. At times campaigners for safe space flaunt their vulnerability and fragility to justify their demand for protection. The imperative of insulating human fragility from the emotional pain caused by offending words and criticisms continued to play a role even at the height of the pandemic. Numerous calls for safe spaces during the pandemic drew attention to the need for a space where they could feel comfortable and conduct their affairs without being judged. “Having a space where LGBTQ people can simply exist in their own skin and experience, without judgment or pressure to hide for the benefit of cisgender, heterosexual people, can be enormously beneficial” wrote one LGBTQ advocate in May 2020.^10^ It is worth noting that statements drawing attention to the benefits of safe spaces during the pandemic invariably praised their non-judgmental ethos.

When I carried out a content analysis of documents calling for safe spaces in 2016–17, I was struck by the regularity with which the avoidance of judgment featured as their key objective.^11^ In effect a safe space provides a quarantine from the threat of judgment. That is why from this perspective, free speech and robust debate are often diagnosed as unsafe and a danger to mental health. Supporters of safe space regard the absence of judgment as one of the most cherished features of this institution. This point is explicitly recognised by many universities who advertise their commitment to the core value of non-judgmentalism. The Student Services Value Statement of St Andrew University promises to “actively reflect” on its “practice to ensure our environment is non-judgemental”.^12^ Universities regularly portray their safe spaces as havens from judgment. “Safe Zone provides an avenue for LGBTQ individuals to be able to identify places and people who are supportive, non-judgmental, and welcoming of open dialogues regarding these issues” declares Montana State University in its advert for its Safe Zone.^13^

As was the case on campuses in the pre-pandemic era, so today a safe space promises to provide a quarantine from the threat of judgment. Judging by the values statements produced by universities and public institutions, contemporary elite culture has become estranged from moral judgment. It frequently communicates the idea that judgment is inherently harmful, and it therefore incites people to quarantine themselves from criticism and judgment.

## The Personalisation of Spaces and Boundaries

In the contemporary world, social distancing and the quest for safe space has acquired a hyper-individualistic form. In recent decades, concern with personal space has been paralleled by anxieties about personal boundaries. The self-help industry has responded to these concerns by publishing advice books and offering guidance on how to protect personal boundaries and maintain distance from others. Helping people protect their personal space through establishment of boundaries has become a flourishing enterprise.

The titles of self-help literature portray the “setting” of a personal boundary as a duty to the self, as in Charles Whitfield’s *Boundaries and Relationships: Knowing, Protecting and Enjoying the Self* (1993). *Anne Katherine’s Where to Draw the Line: How to Set Healthy Boundaries Every Day* (2000) communicates the conviction that drawing lines is a daily ritual. Numerous books, such as Henry Cloud and John Townsend’s *Boundaries in Marriage* (2002), offer advice about how to set boundaries between yourself and others, including (and often, especially) the people that you love. Jennifer Miller and Victoria Lambert’s *How to Draw the Line in Your Head and Home* (2018) aims to provide assistance to readers who need to find ways of establishing personal boundaries in their domestic life. Adelyne Birch’s, *Boundaries After a Pathological Relationship* suggests that it is never too late to establish personal boundaries. A heightened sense of pre-occupation with protecting the self suggests that the defence of space and territory has acquired a hyper-personal form.

Personal boundaries are sometimes referred to as embodied boundaries. This focus on the personal body is accompanied a by a sense of anxiety about how to construct a boundary around the self. Numerous seminars and workshops are devoted to guide individuals to come to terms with their embodied boundary. An invitation to one such workshop asks “where does self end and other begin?” It continues: “How can ‘we’ be a safe experience? Learn about the natural development of ‘boundary’ from a somatic and developmental perspective. This workshop will help therapists work psychophysically with clients around self regulation and relational issues.”^14^ Professional advice is now available on “the hard work of creating healthier boundaries in your day to day life.”^15^

Advice on setting personal boundaries tends to encourage the establishment of hard, inflexible lines. Mark Manson’s *Guide to Strong Relationship Boundaries* advises that, “A person with strong boundaries understands that it’s unreasonable to expect two people to accommodate each other 100 per cent and fulfil every need the other has.”^16^ Manson’s strong boundaries implicitly encourage the psychic and personal distancing of people involved in a relationship. He states that “a person with strong boundaries understands that they may hurt someone’s feelings sometimes, but ultimately they can’t determine how other people feel.” Statements such as this seek to reconcile people to their estrangement from one another, and to protect them from being disappointed with other people.

The call to construct personal boundaries is often self-consciously directed at appealing to the absence of trust in society. Mannheim’s coupling of the imperative of social distancing with mistrust helps illuminate the discussion surrounding personal boundaries. The appeal of boundary setting in the domain of personal relationships is fuelled by the kind of existential anxieties that draw sections of society towards the construction of safe spaces. One commentator poses the question of ‘What Are Boundaries and Why Do I Need Them?’ before answering in the following terms: “All relationships need boundaries. A boundary is an imaginary line that separates me from you. They separate your physical space, your feelings, needs and responsibilities from others… Without boundaries, people may take advantage of you because you haven’t set limits about how you expect to be treated.”^17^ In a different context, appeals to people’s insecurity and anxiety of being taken advantage of, would be characterised as an example of the politics of fear.

A heightened sense of mistrust, even of those who are closest to us incites people to distance themselves from other people. The threat of contamination by a virus like COVID-19 has served as a rhetorical idiom through which human beings and the relationships they enter into are portrayed as polluting. Consequently, terms like pollution and toxicity do not simply pertain to physical phenomena. In recent decades, every close relationship of personal dependence can be diagnosed as a cause of debilitating emotional injury. The toxic metaphor has been extended to describe love relations, friendships and relations at work. The title of the first chapter of Harriet Braiker’s *Lethal Lovers and Poisonous People: How to Protect Your Health from Relationships that Make You Sick*, sums up the book’s attitude to relationships. “Warning: This Relationship May be Harmful to Your Health.”^18^ Florence Isaacs’ *Toxic Friends/ True Friends* expands the use of the toxic metaphor to the domain of friendship.^19^ And *Toxic Emotions at Work* by Peter Frost uses the metaphor to account the emotional distress cause by relations at work.”^20^ The well-known psycho-mystic Deepak Chopra warns against contaminating your body with toxic emotions. The term toxic emotion contains the assumption of being dependent on another person. Detoxification involves breaking free from a relation of dependency through establishing non-negotiable boundaries between you and those closest to you.

Social distancing like the setting of personal boundaries is underpinned by a cultural sensibility oriented towards an aversion to risk and a fear of uncertainty. Social distancing is about setting spatial limits and boundaries. But it is meshed with the impulse to set a psychic distance between the self and others. The demand for advice on where and how to set personal boundaries speaks to the lack of confidence towards the management of uncertainty. One way of avoiding this problem is through the self-conscious setting of personal limits, A contributor to *Psychology Today* explains that, “boundaries can be defined as the limits we set with other people, which indicate what we find acceptable and unacceptable in their behaviour towards us.”^21^ Another counsellor states that “it can be useful to think” about boundaries “as our “limits.”^22^ The call to “know your limits” is invariably linked with the project of defining “your intellectual, emotional, physical, and spiritual boundaries” with “strangers, work colleagues, friends, family, and intimate partners.”^23^

The exhortation to set your personal boundary is often conveyed through warnings about the psychological costs of failing to do so. The failure to set boundaries has also become medicalised. The condition co-dependency is presented as a mental health problem suffered by individuals who fail to set limits about what an individual does for others or allows others to do for them. Borderline personality disorder is often used to refer to people who focus on the problems of others rather than tending to their own needs. The inability to set boundaries is often diagnosed as the outcome of a psychological deficit due to loss of a sense of self where an individual does not see others as fully separate from themselves.

The endeavour of setting personal boundaries coexists with the problematisation of personal space. The exhortation “give me some space!” signifies a demand for a zone that is protected from the physical and emotional intrusion of others. Psychologists, in particular, are keen to offer advice on how to maintain and protect your personal space and avoid encroaching and invading other people’s. Even neuroscience has been mobilised to support the legitimacy of this personal zone. Michael Graziano, author of *The Spaces Between Us* argues that there “really is such thing” as personal space^24^ – it is “a protected zone that provides an invisible spatial scaffold that shapes the way humans interact with one another.”^25^ What psychologists also dub “peripersonal space” is depicted as a buffer zone around the body, which protects the individual from invasive remarks, gestures and physical touches.

The seriousness with which the inviolability of personal space is upheld more than matches calls to protect the integrity of national borders. After noting that “don’t invade my personal space” and “respect my boundaries” are “phrases we hear a lot today”, a reviewer in *National Geographic* asks “are we in danger of becoming too obsessed with the idea of personal space?” His answer is an unequivocal “no” – in fact, “we’re in danger of the opposite!”^26^

One symptom of the insecurity regarding personal boundaries is the politicisation of touching. In 2019, during the course of his campaign for Democratic Party presidential candidate, former Vice President Joseph Biden was frequently accused of getting too physically close to people. Biden was forced on the defensive when two women alleged that he made them feel uncomfortable by coming too close to them and being too familiar. Biden responded by releasing a video in which he reflected on the importance of personal space, noting that “social norms have begun to change” and affirming that the “boundaries of protecting personal space have been reset – and I get it.”^27^

## Social Distancing after the Pandemic

The coronavirus has played an important role in reinforcing the trend towards social distancing and the quest for personal boundaries and safe spaces. Although, it is too early to be certain about how these trends will manifest themselves in the post-pandemic world, it is likely that the heightened sense of insecurity that accompanied the lockdown as well as the institutionalisation of social distancing will reinforce the illusory quest for safe spaces and create a demand for new forms physical and psychic distancing. The causal manner with which Dr., Anthony Fauci, President Trump’s chief advisor on the pandemic, declared that the handshake could become a relic of history in the post coronavirus age is significant in this respect.

It is important to note that the medicalisation of touching and physical contact has for long been promoted by moral entrepreneurs. Many western societies have adopted no-touch rules to govern the relation between adults and children. The advocacy of physical distancing existed for some time before the outbreak of the Covid-19 pandemic. As I explain in my book Why Borders Matter, western society has become obsessed with protecting personal space to the point of encouraging the physical distancing of one another. Psychologists, in particular, are keen to offer advice on how to maintain and protect your personal space and avoid encroaching and invading other people’s. In all these cases it was not public health, but the risks associated with close human contact that encouraged the policing of personal boundaries.

Two years before the outbreak of the coronavirus pandemic, in 2018, the *Oxford English Dictionary* chose toxic as its international word of the year. The *OED*’s word of the year is chosen to reflect “the ethos, mood, preoccupations” of a particular year. This choice and the widespread application of the word toxic to describe human beings and relationships – toxic parents, toxic family, toxic friends, toxic emotions, toxic masculinity – indicates that we no longer need the excuse of a pandemic to reinforce the physical and psychic boundary surrounding our personal life. Somewhere along the line the vision of human relations as polluting has become an integral feature of the contemporary *zeitgeist*.

## Footnotes

1 https://www.kcl.ac.uk/policy-institute/assets/getting-used-to-life-under-lockdown.pdf

2 https://www.dailymail.co.uk/news/article-8349223/Coronaphobia-grips-nation-Britons-fear-lockdown-eased-rapidly.html

3 Karl Mannheim, *Systematic Sociology: An Introduction to the Study of Society*: *Collected Works of Karl Mannheim*, vol. 8 (New York: Routledge [1936] 1957), p. 47.

4 Ibid., p. 48.

5 Anthony Giddens, *Modernity and Self-Identity: Self and Society in the Late Modern Age* (Cambridge: Polity, 1991).

6 Andrew Szasz, *Shopping Our Way to Safety: How We Changed from Protecting the Environment to Protecting Ourselves* (Minneapolis, MN: University of Minnesota Press, 2009.

7 Anthony Fry, *Safe Space: How to Survive in a Threatening World* (New York: Dent, 1987), p. xiv.

8 Ibid., Chapter 7.

9 https://www.wired.com/story/amid-pandemic-geography-returns-with-a-vengeance/

10 https://www.vox.com/identities/21266301/lgbtq-people-queer-spaces-coronavirus-pandemic

11 Frank Furedi, *What’s Happened to The University? A Sociological Exploration of its Infantilization* (New York: Routledge 2017), Chapter 4, “Safe Space”.

12 https://www.st-andrews.ac.uk/media/student-services/documents/VALUES%20&%20BELIEFS%20Graphic%202015%20-%202019.pdf (Accessed 26 June 2020).

13 http://www.montana.edu/counseling/safezone.html . (Accessed 26 June 2020).

14 https://www.eventbrite.com/e/embodied-boundaries-establishing-safety-differentiating-self-from-other-registration-52868112949

15 https://www.facebook.com/events/18-hook-ave-toronto-on-m6p-1t4-canada/embodied-boundaries-workshop-in-toronto/237487486797353/

16 Mark Manson “The Guide to Strong Relationship Boundaries”, https://markmanson.net/boundaries

17 Sharron Martin ‘What Are Boundaries and Why Do I need Them?’ https://livewellwithsharonmartin.com/what-are-boundaries/

18 Harriet Braiker, *Lethal Lovers and Poisonous People: How to Protect Your Relationships That Make You Sick* (Lincoln, NE: iUniverse, 2001).

19 Florence Isaacs, *Toxic Friends/ True Friends*, (New York: William Morrow, 1999).

20 Peter Frost, *Toxic Emotions at Work*, (Cambridge, MA: Harvard Business School Press, 2003).

21 Mariana Bockarova, “4 Ways to Set and Keep Your Personal Boundaries.” In *Psychology Today*, https://www.psychologytoday.com/gb/blog/romantically-attached/201608/4-ways-set-and-keep-your-personal-boundaries

22 Jo Baker, “Knowing your limits. What are boundaries and why are they important?” In *Counselling Directory;*6 November 2018, https://www.counselling-directory.org.uk/counsellor-articles/knowing-your-limits-what-are-boundaries-and-why-are-they-important

23 Bockarova, op. cit.

24 Graziano is cited in Simon Worrall “You Need Your Personal Space-Here’s the Science Why.” In *National Geographic*, 20 January 2018, https://www.nationalgeographic.com/news/2018/01/personal-space-between-us-graziano-peripersonal-dyspraxia/

25 Michael Graziano, M., *The Space Between Us: A Story of Neuroscience, Evolution, and Human Nature* (New York: Oxford University Press, 2018.

26 Simon Worrall, “You Need Your Personal Space-Here’s the Science Why.” In *National Geographic*, 20 January 2018, https://www.nationalgeographic.com/news/2018/01/personal-space-between-us-graziano-peripersonal-dyspraxia/

27 Cited in Benedict Carey “Beyond Biden; How Close is Too Close.” *New York Times*, April 4, 2019.

